# Developing and enhancing biodiversity monitoring programmes: a collaborative assessment of priorities

**DOI:** 10.1111/1365-2664.12423

**Published:** 2015-04-02

**Authors:** Michael J. O. Pocock, Stuart E. Newson, Ian G. Henderson, Jodey Peyton, William J. Sutherland, David G. Noble, Stuart G. Ball, Björn C. Beckmann, Jeremy Biggs, Tom Brereton, David J. Bullock, Stephen T. Buckland, Mike Edwards, Mark A. Eaton, Martin C. Harvey, Mark O. Hill, Martin Horlock, David S. Hubble, Angela M. Julian, Edward C. Mackey, Darren J. Mann, Matthew J. Marshall, Jolyon M. Medlock, Elaine M. O'Mahony, Marina Pacheco, Keith Porter, Steve Prentice, Deborah A. Procter, Helen E. Roy, Sue E. Southway, Chris R. Shortall, Alan J. A. Stewart, David E. Wembridge, Mark A. Wright, David B. Roy

**Affiliations:** ^1^ Centre for Ecology & Hydrology Maclean Building, Benson Lane, Crowmarsh Gifford Wallingford, Oxfordshire OX10 8BB UK; ^2^ British Trust for Ornithology The Nunnery, Thetford Norfolk IP24 2PU UK; ^3^ Conservation Science Group Department of Zoology University of Cambridge Cambridge CB2 3EJ UK; ^4^ JNCC Monkstone House, City Road Peterborough, Cambridgeshire PE1 1JY UK; ^5^ Freshwater Habitats Trust Bury Knowle House, North Place Headington, Oxford OX3 9HY UK; ^6^ Butterfly Conservation Manor Yard, East Lulworth Wareham, Dorset BH20 5QP UK; ^7^ National Trust Heelis, Kemble Drive Swindon, Wiltshire SN2 2NA UK; ^8^ CREEM University of St Andrews The Observatory, Buchanan Gardens St Andrews, Fife KY16 9LZ UK; ^9^ Leaside Carron Lane Midhurst, West Sussex GU29 9LB UK; ^10^ RSPB Centre for Conservation Science The Lodge Sandy, Bedfordshire SG19 2DL UK; ^11^ Department of Environment, Earth and Ecosystems The Open University Walton Hall Milton Keynes MK7 6AA UK; ^12^ 11 Chaucer Road Cambridge, Cambridgeshire CB2 7EB UK; ^13^ Norfolk Biodiversity Information Service County Hall, Martineau Lane Norwich, Norfolk NR1 2SG UK; ^14^ Chrysomelid Recording Scheme 28 St. Mary's Road Eastleigh, Hampshire SO50 6BP UK; ^15^ Amphibian and Reptile Groups of UK (ARGUK) & Amphibian and Reptile Conservation (ARC) 655A Christchurch Road, Boscombe Bournemouth, Dorset BH1 4AP UK; ^16^ Scottish Natural Heritage Silvan House, 231 Corstorphine Road Edinburgh EH12 7AT UK; ^17^ Oxford University Museum of Natural History Parks Road Oxford, Oxfordshire OX1 3PW UK; ^18^ The Wildlife Trusts The Kiln, Waterside, Mather Road Newark, Nottinghamshire NG24 1WT UK; ^19^ Medical Entomology Group Public Health England Porton Down Salisbury, Wiltshire SP4 0JG UK; ^20^ Bumblebee Conservation Trust School of Biological and Environmental Science University of Stirling Stirling FK9 4LA UK; ^21^ The Mammal Society 3 The Carronades, New Road Southampton, Hampshire SO14 0AA UK; ^22^ Natural England Suite D, Unex House, Bourges Boulevard Peterborough PE1 1NG UK; ^23^ British Dragonfly Society c/o Natural England Parkside Court, Hall Park Way Telford, Shropshire TF3 4LR UK; ^24^ Plantlife International 14 Rollestone Street Salisbury SP1 1DX UK; ^25^ Rothamsted Insect Survey Department of Agroecology Rothamsted Research Harpenden, Hertfordshire AL5 2JQ UK; ^26^ School of Life Sciences University of Sussex Falmer, Brighton Sussex BN1 9QG UK; ^27^ People's Trust for Endangered Species 15 Cloisters House, 8 Battersea Park Road London W8 4BG UK; ^28^ Northern Ireland Environment Agency Klondyke Building, Cromac Avenue Belfast County Antrim BT7 2JA UK

**Keywords:** biodiversity, citizen science, monitoring, participatory monitoring, surveillance, survey, volunteer

## Abstract

1. Biodiversity is changing at unprecedented rates, and it is increasingly important that these changes are quantified through monitoring programmes. Previous recommendations for developing or enhancing these programmes focus either on the end goals, that is the intended use of the data, or on how these goals are achieved, for example through volunteer involvement in citizen science, but not both. These recommendations are rarely prioritized.
2. We used a collaborative approach, involving 52 experts in biodiversity monitoring in the UK, to develop a list of attributes of relevance to any biodiversity monitoring programme and to order these attributes by their priority. We also ranked the attributes according to their importance in monitoring biodiversity in the UK. Experts involved included data users, funders, programme organizers and participants in data collection. They covered expertise in a wide range of taxa.
3. We developed a final list of 25 attributes of biodiversity monitoring schemes, ordered from the most elemental (those essential for monitoring schemes; e.g. articulate the objectives and gain sufficient participants) to the most aspirational (e.g. electronic data capture in the field, reporting change annually). This ordered list is a practical framework which can be used to support the development of monitoring programmes.
4. People's ranking of attributes revealed a difference between those who considered attributes with benefits to end users to be most important (e.g. people from governmental organizations) and those who considered attributes with greatest benefit to participants to be most important (e.g. people involved with volunteer biological recording schemes). This reveals a distinction between focussing on aims and the pragmatism in achieving those aims.
5. *Synthesis and applications*. The ordered list of attributes developed in this study will assist in prioritizing resources to develop biodiversity monitoring programmes (including citizen science). The potential conflict between end users of data and participants in data collection that we discovered should be addressed by involving the diversity of stakeholders at all stages of programme development. This will maximize the chance of successfully achieving the goals of biodiversity monitoring programmes.

## Abstract 2

The ordered list of attributes developed in this study will assist in prioritizing resources to develop biodiversity monitoring programmes (including citizen science). The potential conflict between end users of data and participants in data collection that we discovered should be addressed by involving the diversity of stakeholders at all stages of programme development. This will maximize the chance of successfully achieving the goals of biodiversity monitoring programmes.

## Introduction

Biodiversity is changing at an unprecedented rate: many species are declining in abundance (Butchart *et al*. 2010) and there is increasing biotic homogenization across the globe (McKinney & Lockwood 1999). These changes have direct consequences for human well‐being, for example by impacting on the benefits we gain from nature through ecosystem services (Millennium Ecosystems Assessment 2005).

As a result of concern about the changing state of biodiversity, international targets have been agreed with the aim of bringing a reduction in the rates of loss, for example the Convention on Biological Diversity's Aichi Targets (http://www.cbd.int/sp/targets/). Performance against these targets is assessed with currently available biodiversity monitoring information (Baillie, Hilton‐Taylor & Stuart 2004; Butchart *et al*. 2010). However, the currently available information is incomplete (Scholes *et al*. 2008; Pereira *et al*. 2013). Therefore, performance against these targets cannot be adequately assessed unless monitoring, and analysis of data, is enhanced. In addition to these statutory and operational requirements, biodiversity monitoring data are also essential for ecological research (Fisher, Frank & Leggett 2010) and informing conservation management (Whittaker *et al*. 2005; Pereira, Navarro & Martins 2012). Undertaking monitoring of local resources can also empower local stakeholders, including indigenous people in the tropics (Gadgil 2000; Danielsen *et al*. 2011; Pritchard 2013).

Obtaining data on changes in biodiversity, which is typically based on data on the presence and abundance of species (Butchart *et al*. 2010), relies on the availability of participants to make records. If we restrict monitoring to that done by professional ecologists, then data will be limited by their distribution and scarcity, and the availability of funding to employ them (Martin, Blossey & Ellis 2012). Alternatively, engaging non‐professionals (i.e. volunteers) can contribute to the success of long‐term and large‐scale monitoring through their commitment, enthusiasm and geographic spread (Schmeller *et al*. 2009; Danielsen *et al*. 2011; Mackechnie *et al*. 2011; Hochachka *et al*. 2012; Miller‐Rushing, Primack & Bonney 2012), and their role as local stakeholders and resource managers (Danielsen *et al*. 2011; Mant *et al*. 2013). Indeed, considering volunteers in participatory monitoring is an example of ‘citizen science’ which is increasingly being recognized as a credible tool for scientific research and monitoring (Dickinson, Zuckerberg & Bonter 2010; Dickinson & Bonney 2012; Danielsen *et al*. 2014).

Recording natural history by volunteers is an activity that has taken place for a long time; in countries such as the UK, it has flourished for centuries (Preston, Roy & Roy 2012). Different types of recording have different costs and benefits (Tulloch *et al*. 2013b), and recording natural history has been challenged for being ‘curiosity‐driven’, rather than structured and systematic (Lindenmayer & Likens 2010). However, while the data from ‘unstructured’ recording can be more challenging to deal with than structured data, they can produce accurate and statistically rigorous results (Szabo, Fuller & Possingham 2012; Van Strien, van Swaay & Termaat 2013; Isaac *et al*. 2014) which have relevance for academic research, policy and public interest, for example as amply demonstrated in the UK and Republic of Ireland (Asher *et al*. 2001; Preston, Pearman & Dines 2002; Biesmeijer *et al*. 2006; Balmer *et al*. 2013).

Given the need for information on biodiversity change, a key question is how to develop (i.e. begin or enhance) monitoring programmes? A sustainable answer to such a question has to consider the needs both of end users of the data and of the participants who make the records. Currently, there is clear, goal‐oriented advice on the ‘sequence of key steps’ to begin a monitoring programme that is focussed on meeting the needs of end users (Noon 2002; Lindenmayer & Likens 2010; James 2011; Gitzen *et al*. 2012). Separately, guidance has been produced on undertaking monitoring with citizen science, and supporting volunteer participants, so emphasizing engagement with the general public (Tweddle *et al*. 2012; Cornell Lab of Ornithology 2013) and local stakeholders, for example through participatory biodiversity monitoring by indigenous people (Mant *et al*. 2013).

Currently, there is no guidance on considering both the strategic goals *and* the motivations of participants in biodiversity monitoring, yet this is essential to help prioritize resources for developing monitoring programmes. Our aim was to provide such guidance, applicable anywhere in the world, by collaboratively drawing on the breadth of expertise from the UK. We had two objectives: (i) produce a list of attributes to be considered when developing a biodiversity monitoring programme and to order it from the most fundamental to the most aspirational attributes and (ii) identify which attributes were priorities for monitoring in the UK and identify differences in stakeholder perception of these priorities. We followed recommendations in Sutherland *et al*. (2011) and worked collaboratively as a partnership with a wide experience of monitoring biodiversity in the UK.

## Materials and methods

Fifty‐two people were invited to participate in this project. Invited participants were people experienced in monitoring biodiversity in the UK (having strategic oversight or extensive practical experience, acting in a professional or voluntary capacity). They included volunteer experts who run biological recording schemes and societies and coordinate other volunteers to gather species records, academics, representatives from non‐governmental conservation organizations and government agencies. We selected participants to ensure the group had wide taxonomic expertise, from popular groups (such as birds) to those for which skills in identification or sampling are less commonplace (see Appendix S1 in Supporting Information). The process consisted of three tasks (Table 1; detailed in Appendices S2 and S3) and culminated in a workshop held on 22 January 2013. A varying number of ‘respondents’ took part in each task (Table 1); those at the final workshop are authors. All tasks were designed by a subset of the authors (MJOP, SEN, IGH, JP & DBR).

**Table 1:** Summary of the aims and objectives of and respondents to the three tasks of this project to gather expert opinion and address how to develop biodiversity monitoring programmes. 52 people were invited to participate in the tasks, although task 2 was also open to participation by anyone. Participation in tasks 1 and 2 was via email or internet surveys, while participation in task 3 was at a workshop

Task number	Aim	Objective	Number of respondents
1	Produce list of attributes for a biodiversity monitoring programme	Produce a finalized list for consideration in tasks 2 and 3	37 invited participants commenting on an initial list created by MJOP, SEN, IGH, JP & DBR
2	Rank the 10 most important needs for monitoring biodiversity	Identify which attributes are perceived to be the most important needs for monitoring biodiversity in Britain	43 invited participants, plus 119 others responding to the open invitation
3, part 1	Rank all the statements from the most elemental to the most aspirational	Create an ordered list as a basis for discussions in task 3, part 2	17 invited participants
3, part 2	Collaborative ranking of the statements from the most elemental to the most aspirational	Agree on an ordered list of attributes for programmes monitoring biodiversity change which is applicable anywhere in the world, at any scale and for any taxonomic group	36 of the invited participants attending the workshop

### Task 1: Collaboratively Developing a List of Attributes of Biodiversity Monitoring Programmes

We considered individual ‘attributes’ of biodiversity monitoring, that is discrete components or ‘key steps’ in monitoring programmes. An initial list of attributes was produced by a subset of the authors (MJOP, SEN, IGH, JP & DBR) and circulated to all invited participants. Suggestions to improve the list were received and it was revised (Table 1, Appendices S2 and S3) until we had developed a list, agreed by consensus, of the attributes of biodiversity monitoring programmes. The list comprised 24 attributes in total, with one attribute added during task 3.

### Task 2: Assessing the Importance of Attributes

The aim of the second task was to collate opinions on the importance of the attributes for biodiversity monitoring programmes in the UK and understand how this differed by the type of stakeholder. First, participants ranked 10 attributes out of the total of 24, which they considered were the greatest needs or opportunities for developing existing biodiversity monitoring programmes. Respondents were asked to consider their own, current experience, so we could describe variation in the responses in terms of the respondent's affiliation (government organization, non‐governmental organization, recording scheme or society, other or none) and taxonomic focus. This therefore differed from the collaborative approach (Sutherland *et al*. 2011) applied elsewhere in this study. People with more than one affiliation (e.g. as an employee of an organization but also a volunteer recorder) were invited to take part more than once. We also opened the invitation to participate via an announcement at the National Biodiversity Network conference 2012 and promotion by the authors. The ranked needs were scored from ten (most important) to one (least important) using an online survey tool (Survey Monkey: www.surveymonkey.com). For respondents who ranked more than 10 attributes (7%), we only considered their top 10. For those who ranked fewer than 10 statements (12%; all ranked at least five), we scored all those ranked.

Secondly, we tested for differences between respondents’ ranking based on their individual traits. Similar approaches have been used previously, either with the respondents under investigation as authors (Dicks *et al*. 2013) or not (Rudd & Fleishman 2014). The subset of authors who designed this task did not participate. A principal components analysis (PCA) of the ranked attributes was undertaken, and to confirm a lack of bias, it was repeated with different combinations of respondents (Appendix S4). The results were clustered with *k*‐means clustering into an optimum number of clusters, as identified with the gap statistic (Tibshirani, Walther & Hastie 2001) in the package ‘NbClust’ (Charrad *et al*. 2013) in R version 3.0.2. The association of clusters with respondents’ traits was tested with *G* ^2^ tests. *Post hoc* partitioning using *G* ^2^ tests identified the importance of specific trait values (Agresti 2013). The traits were as follows: affiliation, taxonomic expertise (vertebrates, invertebrates, vascular plants, bryophytes/lichens/fungi or all) and the regularity of the reporting of change in their taxon of interest (annual, less than annual or none; Appendix S1).

### Task 3: Collaboratively Ordering the List of Attributes of a Biodiversity Monitoring Programme

In our final task, we (the authors) ordered the attributes of a programme to monitor biodiversity change from the most elemental (the essential, basic attributes needed to monitor change) to the most aspirational (the desirable aspects that would add value to a monitoring programme but are not necessarily expected to be achieved). Typically, more elemental attributes have to be achieved in order to achieve the more aspirational attributes.

We collaboratively ordered the attributes at a face‐to‐face workshop. We began with an ordered list (part 1 of task 3; details in Appendix S3) and then discussed further changes until consensus was reached, as indicated by votes at each decision via a show of hands (following Sutherland *et al*. 2011). Throughout, we emphasized that statements could be split, aggregated, reworded or omitted and new ones could be added and that the final list should have widespread applicability.

## Results

Of the 52 invited participants in this collaborative project, more than two‐thirds of people participated in each task (Table 1). They represented wide taxonomic experience. About one‐sixth were from government agencies, one‐sixth were from universities or research institutes, one‐third were from non‐governmental environmental organizations, and one‐third were from volunteer‐led biological recording schemes and societies (Appendix S1).

### Task 1: Obtaining a List of Attributes of a Biodiversity Monitoring Programme

**Figure 1:**
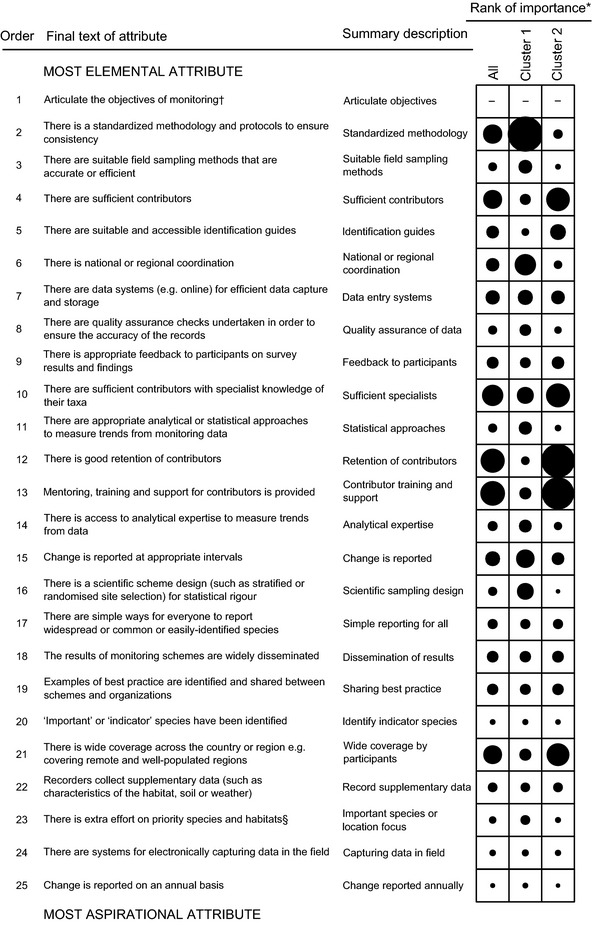
The attributes of a monitoring programme, ordered from most elemental (top) to most aspirational (bottom). The circle size indicates the average rank that respondents gave when they ranked the ten most important attributes according to their own perspective (larger circles being greater needs). *Normalized average rank given by respondents for the importance of each attribute, with larger circles indicating attributes that were classed as more important. †The first attribute was added after task 2 was completed, but is included here for completeness. §This attribute was separated into species and habitats in this survey. Placing effort on priority habitats was scored extremely low, and these scores are not presented here.

We collaboratively produced a list of 25 attributes covering all the components of relevance to biodiversity monitoring programmes (Fig. 1; Appendix S3). Few changes, apart from minor rewording, were made to the list during the remaining tasks.

### Task 2: Clustering Respondents’ Opinions on the Current Needs for Biodiversity Monitoring Programmes

The majority of those who took part in task 2 answered the survey once (but, to represent their different affiliations, three answered it twice and one answered it three times). In addition, 119 people took part by responding to our open invitation. The overall results were similar with all the different subsets of participants (invited participants, non‐authors and everyone; Appendix S4; full data in Appendix S5), indicating that the additional respondents were not a biased subset, and so here we have used the complete data set.

**Figure 2:**
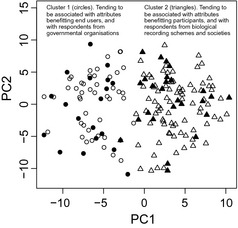
The principal components analysis of responses in which a participant with experience of monitoring biodiversity in the UK ranked the top 10 attributes for improved monitoring of biodiversity change. Two clusters were identified in the responses (circles and triangles), showing strong association with the first principal component (PC1; Table 2). Filled symbols represent responses from the invited participants; others are from those responding to the open invitation to be involved.

We found two distinct clusters in the multivariate analysis of people's ranked attributes, based on their position on the first principal component (PC1; Fig. 2). PC1 described the distinction between cluster 1: attributes that primarily benefitted end users (e.g. standardized protocols, scientific sampling design and national coordination: negative values) and cluster 2: attributes that primarily benefit participants (e.g. retaining and training volunteers: positive values). There was a significant association between cluster membership and affiliation (*G* ^2^_4_ **=* *10·7, *P *=* *0·03) and the taxonomic group for which they had expertise (*G* ^2^_5_ **=* *11·7, *P *=* *0·04), but not the current regularity of reporting for the programme in which they had expertise (*G* ^2^_3_ **=* *3·6, *P *=* *0·17). *Post hoc* tests revealed that respondents affiliated to government organizations were significantly more likely to be in cluster 1, and those with experience in recording invertebrate groups were more likely to be in cluster 2. All other differences were non‐significant. The overall finding of a difference between end users of data and participants in monitoring is of wide relevance, even though the specific findings are relevant to UK monitoring.

### Task 3: Ordering Attributes of a Programme to Monitor Biodiversity Change from Elemental to Aspirational

During the workshop, we collaboratively ordered the attributes from the most elemental to the most aspirational (Fig. 1; details in Appendix S3). Our discussion also resulted in the unanimous decision to add the statement ‘articulate the objectives of the monitoring’ as the most elemental attribute. A few other changes in wording were proposed, and they were accepted or rejected following discussion among participants (Appendix S3). Matching the ranked priorities of attributes (task 2) to their position in this ordered list shows that the attributes deemed to be important for developing UK monitoring programmes are distributed across the range from elemental to aspirational attributes (Fig. 1). This indicates the breadth of our participants’ experience (from well‐established to nascent monitoring programmes) and so gives us confidence that our results can be applied to monitoring anywhere.

## Discussion

There are many different attributes, or components, of biodiversity monitoring programmes. Previously, authors have made recommendations about attributes most applicable to programme organizers, or most beneficial to participants (e.g. in citizen science). However, when considering how to develop (i.e. establish or enhance) programmes, it is important to consider the complete set of attributes, as we have here. We found that the attributes that different people regard as important are broadly separated into those primarily benefitting end users and those primarily benefitting contributors (Table 2; Fig. 1). This reveals a potential conflict between different stakeholders that needs to be reconciled. Both types of attribute occurred throughout the ordered list from elemental to aspirational attributes.

**Table 2:** The attributes of biodiversity monitoring programmes, the primary beneficiary of each attribute and the correlation of the individual respondent's ranked importance of these attributes with the first component from the principal components analysis (loading on PC1)

Summary description of attribute^a^	Primary beneficiary	Loading on PC1 for all data
Standardized methodology	End users + Participants	−0·50
Scientific sampling design	End users	−0·26
National or regional coordination	End users + Participants	−0·21
Suitable field sampling methods	End users + Participants	−0·15
Change is reported	End users	−0·13
Statistical approaches	End users	−0·12
Analytical expertise	End users	−0·10
Data entry systems	Participants	−0·09
Important species focus	End users	−0·07
Quality assurance of data	End users	−0·06
Change reported annually	End users	−0·03
Record supplementary data	End users	−0·03
Simple reporting for all	Participants	−0·03
Capturing data in field	Participants	−0·02
Dissemination of results	End users	−0·02
Identify indicator species	End users	0·01
Important location focus	End users	0·02
Sharing best practice	End users + Participants	0·02
Feedback to participants	Participants	0·04
Identification guides	Participants	0·15
Sufficient specialists	Participants	0·15
Better spatial coverage	End users	0·24
Wide coverage by contributors	End users + Participants	0·28
Contributor training and support	Participants	0·39
Retention of contributors	Participants	0·47

a The full description of each attribute is given in Fig. 1.

In our experience, many biodiversity monitoring programmes involving volunteers have developed incrementally by beginning at small scales and, as their capacity grows, developing in more aspirational ways. We have collaboratively formalized this in a list of attributes ordered from elemental to aspirational attributes. This will help inform the prioritization of resources to support and develop monitoring programmes. There will be variation in the implementation of monitoring, especially comparing developing with developed countries (Danielsen *et al*. 2003), although we believe that the general lessons learnt here remain globally applicable.

### The Ordered List of Attributes of a Biodiversity Monitoring Programme

The ordered list of attributes, from elemental to aspirational attributes (Fig. 1), was derived from the collaboration of experts with a wide range of expertise. Aspirational attributes were those desirable attributes that might be achieved once elemental attributes were in place and as resources permit. Our list included all the 25 attributes that we considered relevant to biodiversity monitoring programmes. These included: having clarity about the end use of the data through to effective motivation of participants (which often has been overlooked by those with a focus on end users of the data). Considering the motivations of participants is especially important when engaging with volunteers, which is likely to be useful and beneficial for biodiversity monitoring programmes (Schmeller *et al*. 2009; Mackechnie *et al*. 2011; Hochachka *et al*. 2012; Danielsen *et al*. 2014).

Our ordered list could be used in two main ways:

1. In a gap analysis of existing programmes. Where there is currently a biodiversity monitoring programme, this could be used as a checklist to reassess priorities, with priority given to the most elemental (top‐ranked) attributes that are not adequately fulfilled. This could then be used by (i) organizers investing in the development of their own monitoring programmes and (ii) funders who are seeking to make the most cost‐effective investments across a range of programmes.
2. When planning development of a biodiversity monitoring programme. Where a biodiversity monitoring programme is to be developed, for example for a taxonomic group in a particular region, it could be used as a checklist to clarify objectives and help justify the investment of resources to support the programme.

This framework is applicable in different geographic regions and ecosystems, over varying time‐scales and with different mixes of professional and volunteer participants because, although the experts were all from the UK, they covered a vast breadth of practical experience in monitoring (not just popular taxonomic groups). However, the challenge of fulfilling each attribute will vary by the type of programme (e.g. for those employing professional surveyors, participant recruitment will be less challenging than when working with volunteers), and the emphasis placed on each one may vary according to the aim of the programme (Lindenmayer & Likens 2010; Tulloch *et al*. 2013b), which may be influenced by geographic region, ecosystem, social context and taxonomic scope. Also when working with local people, especially in developing countries, it is valuable to develop participatory biodiversity monitoring and build upon local knowledge and existing community‐based monitoring (Gadgil 2000; Danielsen *et al*. 2003; Mant *et al*. 2013), especially because they affect and manage patterns of local resource use (Pritchard 2013).

Of course, this framework cannot arbitrate on trade‐offs between the costs and benefits of investing in different attributes, but it does provide an objective starting point for making these decisions.

### The Attributes of Biodiversity Monitoring Programmes

We concluded, as have others, that the most elemental attribute for monitoring biodiversity is having clearly articulated aims (Noon 2002; Beever 2006; Lindenmayer & Likens 2010; Gitzen *et al*. 2012; Danielsen *et al*. 2014) as demonstrated by carefully designed monitoring schemes (e.g. Risely *et al*. 2013) and this is also important for successful citizen science projects (Tweddle *et al*. 2012; Cornell Lab of Ornithology 2013). Having clear aims implies the need to determine statistical power to detect change and to critically assess trade‐offs, for example investment in supporting (professional or volunteer) participants vs. visiting more sites more frequently (Gitzen *et al*. 2012). Notwithstanding this we note, from the experience of unstructured recording in the UK, that much has been gained through development from volunteer enthusiasm rather than beginning with a structured master plan: it has allowed us to discover changes to biodiversity, far beyond what was originally known when data collection began (Thomas *et al*. 2004; Biesmeijer *et al*. 2006).

Volunteers’ time and commitment are key to monitoring biodiversity unless long‐term funding streams are available to employ surveyors. However, volunteers have diverse motivations for participating, and motivations can change as involvement continues and progresses (Ellis & Waterton 2004; Rotman *et al*. 2012). One disadvantage of working with volunteers is that if given a free choice, they are likely to be highly selective in their choice of survey locations (Gregory *et al*. 2004; Tulloch *et al*. 2013a). This explains the emphasis placed by some organizers on systematic scheme design (Newson *et al*. 2005; Gitzen *et al*. 2012; Ferrer‐Paris *et al*. 2013). However, demanding too much of volunteers may reduce levels of participation, and such schemes need to take account of the socio‐economic realities and varying technical capabilities of local people (professionals and volunteers). Developing monitoring schemes with volunteer participants inevitably involves a compromise between an ideal statistical design and ensuring adequate participation to meet the programme goals (Brereton *et al*. 2010; Balmer *et al*. 2013).

One frequently adopted way of focussing monitoring is to target ‘indicator’ species (Landres, Verner & Thomas 1988; Danielsen *et al*. 2011). This can permit efficient sampling, for example assessing against legislative targets (Jongman *et al*. 2013), and is particularly useful where accessible and affordable identification tools are lacking (Ahrends *et al*. 2011). However, the effectiveness of simplified assemblages to truly represent biodiversity or ecosystem change is rarely tested or understood (Landres, Verner & Thomas 1988; McGeogh 1998; Carignan & Villard 2002), hence our caution in recommending indicators as a priority for biodiversity monitoring, and the same concerns apply to focussing on species of conservation concern or which contribute most to ecosystem function. In contrast, while wide species coverage ‘acknowledges the multi‐scale nature and complexity of ecosystems’ (Beever 2006), the risk is that the goals of monitoring become too vague or unachievable.

One of the potential challenges in a monitoring programme is the adequacy of suitable sampling methodology. Although keeping a standard methodology throughout the programme is important, there is also value in being able to incorporate innovative new techniques. Many new sampling methodologies show great promise, for example acoustic surveys (Blumstein *et al*. 2011) and surveying with environmental DNA (Ficetola *et al*. 2008).

### Generalizing About the Current Needs for Monitoring Biodiversity

In assessing the important priorities to enhance biodiversity monitoring in the UK, we found that people tended to emphasize one of two sets of needs. First, there were those who prioritized those attributes of monitoring programmes which give primary benefit to end users of the data, for example having a scientific sampling design (Table 2; Fig. 1). The objectives of these people were focussed on the end goals. People affiliated to governmental organizations, that is end users of the data and funders, were most likely to be in this group. Secondly, there were those who emphasized the needs of volunteers as being most important, for example their recruitment, training and support (Table 2; Fig. 1). The objectives of these people were focussed on participants. Respondents with expertise in monitoring invertebrates were most likely to be in this group, probably because there are fewer recorders of most invertebrates than mammals or birds, so maximizing the recruitment and retention of participants is vital. Importantly, both types of attribute occurred throughout the ordered list from elemental to aspirational attributes, so both types need to be considered however well‐developed the programme is.

For this study, the experts in biodiversity monitoring in the UK gave personal opinions (task 2), in addition to sharing their expertise (tasks 1 and 3). Dicks *et al*. (2013) also considered these two types of participation, but Rudd & Fleishman (2014) separated them. These results reveal the tension between a focus on aims and the pragmatism in achieving those aims. In other words, the motivations of participants who provide the data and end users of the data may be very different (Danielsen *et al*. 2003; Ellis & Waterton 2004; Rotman *et al*. 2012). This highlights the value of dialogue between all the different stakeholders in biodiversity monitoring to resolve potential conflict, as demonstrated by the exemplars of volunteer involvement (i.e. citizen science) in the UK in successful scientifically rigorous long‐term monitoring programmes undertaken by volunteers (Balmer *et al*. 2013; Botham *et al*. 2013; Risely *et al*. 2013).

We predict that participatory monitoring will continue to expand, and policymakers and researchers will increasingly value such data (Danielsen *et al*. 2014). Indeed, in the UK a cultural shift at national policy level has meant that citizen science is now recognized as a potentially effective way of gathering large‐scale information on the impacts of environmental change across a wide range of taxa (Department for Environment Food & Rural Affairs 2011). This change has intensified strategic thinking about achieving aims through careful objective‐driven scheme design. However, participants’ needs must also be considered from the inception of projects, especially where participants are volunteers. For maximum effectiveness, those who focus on end use of the data must consider the needs of participants, while those who focus on participants’ needs must consider the aims and goals of the programme. We recommend that this is best achieved by communication and partnership across stakeholders at all stages in the development and enhancement of biodiversity monitoring programmes.

## Data accessibility

Data are uploaded as online supporting information.

## Supporting information


**Appendix S1.** The composition of the invited participants and additional acknowledgements.
**Appendix S2.** Instructions given to participants in each of the tasks.
**Appendix S3.** The final list of attributes and how they changed through the tasks.
**Appendix S4.** Comparison of the data set with all respondents to that with only the invited participants.

**Appendix S5.** The dataset comprising, for each respondent, the ranks of the attributes that were considered to be the 10 greatest needs or opportunities for developing existing biodiversity monitoring programmes in the UK.

## References

[jpe12423-bib-0001] Agresti, A. (2013) Categorical Data Analysis, 3rd edn John Wiley & Sons, Hoboken, New Jersey.

[jpe12423-bib-0002] Ahrends, A. , Rahbek, C. , Bulling, M.T. , Burgess, N.D. , Platts, P.J. , Lovett, J.C. *et al* (2011) Conservation and the botanist effect. Biological Conservation, 144, 131–140.

[jpe12423-bib-0003] Asher, J. , Warren, M. , Fox, R. , Harding, P. , Jeffcoate, G. & Jeffcoate, S. (2001) The Millenium Atlas of Butterflies in Britain and Ireland. Oxford University Press, Oxford.

[jpe12423-bib-0004] Baillie, J.E.M. , Hilton‐Taylor, C. & Stuart, S.N. (2004) IUCN Red List of Threatened Species. A Global Species Assessment. IUCN, IUCN, Gland, Switzerland and Cambridge, UK.

[jpe12423-bib-0005] Balmer, D. , Gillings, S. , Caffrey, B. , Swann, B. , Downie, I. & Fuller, R. (2013) Bird Atlas 2007–11: The Breeding and Wintering Birds of Britain and Ireland. British Trust for Ornithology, Thetford, UK.

[jpe12423-bib-0006] Beever, E.A. (2006) Monitoring biological diversity: strategies, tools, limitations, and challenges. Northwestern Naturalist, 87, 66–79.

[jpe12423-bib-0007] Biesmeijer, J.C. , Roberts, S.P.M. , Reemer, M. , Ohlemüller, R. , Edwards, M. , Peeters, T. *et al* (2006) Parallel declines in pollinators and insect‐pollinated plants in Britain and the Netherlands. Science, 313, 351–354. 16857940 10.1126/science.1127863

[jpe12423-bib-0008] Blumstein, D.T. , Mennill, D.J. , Clemins, P. , Girod, L. , Yao, K. , Patricelli, G. *et al* (2011) Acoustic monitoring in terrestrial environments using microphone arrays: applications, technological considerations and prospectus. Journal of Applied Ecology, 48, 758–767.

[jpe12423-bib-0009] Botham, M.S. , Brereton, T.M. , Middlebrook, I. , Randle, Z. & Roy, D.B. (2013) United Kingdom Butterfly Monitoring Scheme Report for 2012. Centre for Ecology & Hydrology, Wallingford, Oxfordshire.

[jpe12423-bib-0010] Brereton, T.M. , Cruickshanks, K.L. , Risely, K. , Noble, D.G. & Roy, D.B. (2010) Developing and launching a wider countryside butterfly survey across the United Kingdom. Journal of Insect Conservation, 15, 279–290.

[jpe12423-bib-0011] Butchart, S.H.M. , Walpole, M. , Collen, B. , van Strien, A. , Scharlemann, J.P.W. , Almond, R.E.A. *et al* (2010) Global biodiversity: indicators of recent declines. Science, 328, 1164–1168. 20430971 10.1126/science.1187512

[jpe12423-bib-0012] Carignan, V. & Villard, M.‐A. (2002) Selecting indicator species to monitor ecological integrity: a review. Environmental Monitoring and Assessment, 78, 45–61. 12197640 10.1023/a:1016136723584

[jpe12423-bib-0013] Charrad, M. , Ghazzali, N. , Boiteau, V. & Niknafs, A. (2013) NbClust: An examination of indices for determining the number of clusters. Version 1.4.

[jpe12423-bib-0014] Cornell Lab of Ornithology (2013) Citizen Science Toolkit, http://www.birds.cornell.edu/citscitoolkit/toolkit

[jpe12423-bib-0015] Danielsen, F. , Mendoza, M.M. , Alviola, P. , Balete, D.S. , Enghoff, M. , Poulsen, M.K. & Jensen, A.E. (2003) Biodiversity monitoring in developing countries: what are we trying to achieve? Oryx, 37, 407–409.

[jpe12423-bib-0016] Danielsen, F. , Skutsch, M. , Burgess, N.D. , Jensen, P.M. , Andrianandrasana, H. , Karky, B. *et al* (2011) At the heart of REDD+: a role for local people in monitoring forests? Conservation Letters, 4, 158–167.

[jpe12423-bib-0017] Danielsen, F. , Pirhofer‐Walzl, K. , Adrian, T.P. , Kapijimpanga, D.R. , Burgess, N.D. , Jensen, P.M. *et al* (2014) Linking public participation in scientific research to the indicators and needs of international environmental agreements. Conservation Letters, 7, 12–24.

[jpe12423-bib-0018] Department for Environment Food & Rural Affairs (2011) The Natural Choice: Securing the Value of Nature. The Stationery Office Limited, London, UK.

[jpe12423-bib-0019] Dickinson, J.L. & Bonney, R. (2012) Citizen Science: Public Participation in Environmental Research. Cornell University Press, Ithaca.

[jpe12423-bib-0020] Dickinson, J.L. , Zuckerberg, B. & Bonter, D.N. (2010) Citizen science as an ecological research tool: challenges and benefits. Annual Review of Ecology, Evolution, and Systematics, 41, 149–172.

[jpe12423-bib-0021] Dicks, L.V. , Abrahams, A. , Atkinson, J. , Biesmeijer, J. , Bourn, N. , Brown, C. *et al* (2013) Identifying key knowledge needs for evidence‐based conservation of wild insect pollinators: a collaborative cross‐sectoral exercise. Insect Conservation and Diversity, 6, 435–446.

[jpe12423-bib-0022] Ellis, R. & Waterton, C. (2004) Environmental citizenship in the making: the participation of volunteer naturalists in UK biological recording and biodiversity policy. Science and Public Policy, 31, 95–105.

[jpe12423-bib-0023] Ferrer‐Paris, J.R. , Rodríguez, J.P. , Good, T.C. , Sánchez‐Mercado, A.Y. , Rodríguez‐Clark, K.M. , Rodríguez, G.A. & Solís, A. (2013) Systematic, large‐scale national biodiversity surveys: NeoMaps as a model for tropical regions. Diversity and Distributions, 19, 215–231.

[jpe12423-bib-0024] Ficetola, G.F. , Miaud, C. , Pompanon, F. & Taberlet, P. (2008) Species detection using environmental DNA from water samples. Biology Letters, 4, 423–425. 18400683 10.1098/rsbl.2008.0118 PMC2610135

[jpe12423-bib-0025] Fisher, J.A.D. , Frank, K.T. & Leggett, W.C. (2010) Dynamic macroecology on ecological time‐scales. Global Ecology and Biogeography, 19, 1–15.

[jpe12423-bib-0026] Gadgil, M. (2000) People's biodiversity registers: lessons learnt. Environment, Development and Sustainability, 2, 323–332.

[jpe12423-bib-0027] Gitzen, R.A. , Millspaugh, J.J. , Cooper, A.B. & Licht, D.S. (eds). (2012) Design and Analysis of Long‐Term Ecological Monitoring Studies. Cambridge University Press, Cambridge.

[jpe12423-bib-0028] Gregory, R.D. , van Strien, A. , Vorisek, P. , Meyling, A.W.G. , Noble, D.G. , Foppen, R.P.B. & Gibbons, D.W. (2004) Developing indicators for European birds. Philosophical Transactions of the Royal Society B: Biological Sciences, 360, 269–288. 10.1098/rstb.2004.1602 PMC1569455 15814345

[jpe12423-bib-0029] Hochachka, W.M. , Fink, D. , Hutchinson, R.A. , Sheldon, D. , Wong, W.‐K. & Kelling, S. (2012) Data‐intensive science applied to broad‐scale citizen science. Trends in Ecology & Evolution, 27, 130–137. 22192976 10.1016/j.tree.2011.11.006

[jpe12423-bib-0030] Isaac, N.J.B. , van Strien, A.J. , August, T.A. , de Zeeuw, M.P. & Roy, D.B. (2014) Statistics for citizen science: extracting signals of change from noisy ecological data. Methods in Ecology and Evolution, 5, 1052–1060.

[jpe12423-bib-0031] James, T. (2011) Running a Biological Recording Scheme or Survey. NBN Trust, Nottingham.

[jpe12423-bib-0032] Jongman, R.H.G. , Henle, K. , Bauch, B. , Auliya, M. , Külvik, M. , Pe'er, G. , Schmeller, D.S. & Framstad, E. (2013) Priorities for biodiversity monitoring in Europe: a review of supranational policies and a novel scheme for integrative prioritization. Ecological Indicators, 33, 5–18.

[jpe12423-bib-0033] Landres, P.B. , Verner, J. & Thomas, J.W. (1988) Ecological uses of vertebrate indicator species: a critique. Conservation Biology, 2, 316–326.

[jpe12423-bib-0034] Lindenmayer, D.B. & Likens, G.E. (2010) The science and application of ecological monitoring. Biological Conservation, 143, 1317–1328.

[jpe12423-bib-0035] Mackechnie, C. , Maskell, L. , Norton, L. & Roy, D. (2011) The role of “Big Society” in monitoring the state of the natural environment. Journal of Environmental Monitoring, 13, 2687–2691. 21879098 10.1039/c1em10615e

[jpe12423-bib-0036] Mant, R. , Swan, S. , Bertzky, M. & Miles, L. (2013) Participatory Biodiversity Monitoring: Considerations for National REDD+ Programmes. Prepared by UNEP‐WCMC Cambridge, UK; and SNV REDD+, Ho Chi Minh City, Vietnam.

[jpe12423-bib-0037] Martin, L.J. , Blossey, B. & Ellis, E. (2012) Mapping where ecologists work: biases in the global distribution of terrestrial ecological observations. Frontiers in Ecology and the Environment, 10, 195–201.

[jpe12423-bib-0038] McGeogh, M.A. (1998) The selection, testing and application of terrestrial insects as indicators. Biological Review, 73, 181–201.

[jpe12423-bib-0039] McKinney, M.L. & Lockwood, J.L. (1999) Biotic homogenization: a few winners replacing many losers in the next mass extinction. Trends in Ecology & Evolution, 14, 450–453. 10511724 10.1016/s0169-5347(99)01679-1

[jpe12423-bib-0040] Millennium Ecosystems Assessment (2005) Ecosystems and Human Well‐Being: Biodiversity Synthesis. World Resources Institute, Washington D.C.

[jpe12423-bib-0041] Miller‐Rushing, A. , Primack, R. & Bonney, R. (2012) The history of public participation in ecological research. Frontiers in Ecology and the Environment, 10, 285–290.

[jpe12423-bib-0042] Newson, S.E. , Woodburn, R.J.W. , Noble, D.G. , Baillie, S.R. & Gregory, R.D. (2005) Evaluating the Breeding Bird Survey for producing national population size and density estimates. Bird Study, 52, 42–54.

[jpe12423-bib-0043] Noon, B.R. (2002) Conceptual issues in monitoring ecological resources Monitoring Ecosystems: Interdisciplinary Approaches for Evaluating Ecoregional Initiatives (eds Busch D.E. & Trexler J.C.), pp. 27–72. Island Press, Washington, DC.

[jpe12423-bib-0045] Pereira, H.M. , Navarro, L.M. & Martins, I.S. (2012) Global biodiversity change: the bad, the good, and the unknown. Annual Review of Environment and Resources, 37, 25–50.

[jpe12423-bib-0046] Pereira, H.M. , Ferrier, S. , Walters, M. , Geller, G.N. , Jongman, R.H.G. , Scholes, R.J. *et al* (2013) Essential biodiversity variables. Science, 339, 277–278. 23329036 10.1126/science.1229931

[jpe12423-bib-0047] Preston, C.D. , Pearman, D.A. & Dines, T.D. (2002) New Atlas of the British & Irish Flora: An Atlas of the Vascular Plants of Britain, Ireland, the Isle of Man and the Channel Islands. Oxford University Press, Oxford.

[jpe12423-bib-0048] Preston, C.D. , Roy, D.B. & Roy, H.E. (2012) What have we learnt from 50 years of biological recording? British Wildlife, 24, 97–106.

[jpe12423-bib-0049] Pritchard, D.J. (2013) Community‐based biodiversity monitoring in Mexico: current status, challenges, and future strategies for collaboration with scientists Community Action for Conservation: Mexican Experiences (eds Porter‐Bolland L., Ruiz‐Mallén I., Camacho‐Benavides C. & McCandless S.R.), pp. 135–157. Springer New York, New York, NY.

[jpe12423-bib-0050] Risely, K. , Massimino, D. , Newson, S.E. , Eaton, M.A. , Musgrove, A.J. , Noble, D.G. , Procter, D. & Baillie, S.R. (2013) The Breeding Bird Survey 2012. BTO Research Report 645. British Trust for Ornithology, Thetford, UK.

[jpe12423-bib-0051] Rotman, D. , Preece, J. , Hammock, J. , Procita, K. , Hansen, D. , Parr, C. , Lewis, D. & Jacobs, D. (2012) Dynamic changes in motivation in collaborative citizen‐science projects. *Proceedings of the ACM 2012 Conference on Computer Supported Cooperative Work*, 217.

[jpe12423-bib-0052] Rudd, M.A. & Fleishman, E. (2014) Policymakers’ and scientists’ ranks of research priorities for resource‐management policy. BioScience, 64, 219–228.

[jpe12423-bib-0053] Schmeller, D.S. , Henry, P.‐Y. , Julliard, R. , Gruber, B. , Clobert, J. , Dziock, F. *et al* (2009) Advantages of volunteer‐based biodiversity monitoring in Europe. Conservation Biology, 23, 307–316. 19183201 10.1111/j.1523-1739.2008.01125.x

[jpe12423-bib-0054] Scholes, R.J. , Mace, G.M. , Turner, W. , Geller, G.N. , Jurgens, N. , Larigauderie, A. , Muchoney, D. , Walther, B.A. & Mooney, H.A. (2008) Toward a global biodiversity observing system. Science, 321, 1044–1045. 18719268 10.1126/science.1162055

[jpe12423-bib-0055] Sutherland, W.J. , Fleishman, E. , Mascia, M.B. , Pretty, J. & Rudd, M.A. (2011) Methods for collaboratively identifying research priorities and emerging issues in science and policy. Methods in Ecology and Evolution, 2, 238–247.

[jpe12423-bib-0056] Szabo, J.K. , Fuller, R.A. & Possingham, H.P. (2012) A comparison of estimates of relative abundance from a weakly structured mass‐participation bird atlas survey and a robustly designed monitoring scheme. Ibis, 154, 468–479.

[jpe12423-bib-0057] Thomas, J.A. , Telfer, M.G. , Roy, D.B. , Preston, C.D. , Greenwood, J.J.D. , Asher, J. , Fox, R. , Clarke, R.T. & Lawton, J.H. (2004) Comparative losses of British butterflies, birds, and plants and the global extinction crisis. Science, 303, 1879–1881. 15031508 10.1126/science.1095046

[jpe12423-bib-0058] Tibshirani, R. , Walther, G. & Hastie, T. (2001) Estimating the number of clusters in a data set via the gap statistic. Journal of the Royal Statistical Society B, 63, 411–423.

[jpe12423-bib-0059] Tulloch, A.I.T. , Mustin, K. , Possingham, H.P. , Szabo, J.K. & Wilson, K.A. (2013a) To boldly go where no volunteer has gone before: predicting volunteer activity to prioritize surveys at the landscape scale. Diversity and Distributions, 19, 465–480.

[jpe12423-bib-0060] Tulloch, A.I.T. , Possingham, H.P. , Joseph, L.N. , Szabo, J. & Martin, T.G. (2013b) Realising the full potential of citizen science monitoring programs. Biological Conservation, 165, 128–138.

[jpe12423-bib-0061] Tweddle, J.C. , Robinson, L.D. , Pocock, M.J.O. & Roy, H.E. (2012) Guide to Citizen Science: Developing, Implementing and Evaluating Citizen Science to Study Biodiversity and the Environment in the UK. Natural History Museum and NERC Centre for Ecology & Hydrology for UK‐EOF, London, UK.

[jpe12423-bib-0062] Van Strien, A.J. , van Swaay, C.A.M. & Termaat, T. (2013) Opportunistic citizen science data of animal species produce reliable estimates of distribution trends if analysed with occupancy models. Journal of Applied Ecology, 50, 1450–1458.

[jpe12423-bib-0063] Whittaker, R.J. , Araújo, M.B. , Jepson, P. , Ladle, R.J. , Watson, J.E.M. & Willis, K.J. (2005) Conservation biogeography: assessment and prospect. Diversity and Distributions, 11, 3–23.

