# Supplementary material for: Developing and enhancing biodiversity monitoring programmes: a collaborative assessment of priorities
Source: J Appl Ecol. 2015 Apr 2;52(3):686–95. doi: 10.1111/1365-2664.12423 (PMC5008152; doi:10.1111/1365-2664.12423)
Supplement: Supplementary file 1 — Appendix S1. The composition of the invited participants and additional acknowledgements. Appendix S2. Instructions given to participants in each of the tasks. Appendix S3. The final list of attributes and how they changed through the tasks. Appendix S4. Comparison of the data set with all respondents to that with only the invited participants. [file JPE-52-686-s001.docx]

## Appendix S1:

**Table S1.** The composition of the invited participants (n=52) and additional acknowledgements.

| Participant’s trait | Levels | Number of people | Current frequency of reporting* |
| --- | --- | --- | --- |
| Affiliation | University/research institute | 8 | n/a |
|  | Governmental organisation | 10 | n/a |
|  | Non-governmental environmental organisation | 18 | n/a |
|  | Biological recording scheme or society | 16 | n/a |
| Taxonomic expertise | Fungi | 1 | None |
|  | Lichens | 1 | None |
|  | Bryophytes | 1 | None |
|  | Plants | 2 | Some |
|  | Insects: Coleoptera | 3 | Coccinellidae: some; all others none |
|  | Insects: Diptera | 3 | Syrphidae: some; all others: none |
|  | Insects: Hymenoptera | 2 | Some |
|  | Insects: Lepidoptera | 3 | Butterflies: annual; moths: some |
|  | Insects: others | 4 | Odonata: some |
|  | Non-insect invertebrates | 2 |  |
|  | Amphibians and reptiles | 2 | None |
|  | Birds | 4 | Annual |
|  | Mammals | 2 | Bats: annual; others: some |
|  | No specific expertise | 22 | n/a |

* The current frequency of reporting is an attribute that we assigned to taxonomic groups with which each respondent had expertise. ‘Some’ is reporting less frequent than annually.

In addition to the authors of this paper, we thank the following for their participation: Derek Schafer (British Mycological Society), Kevin Walker (Botanical Society for Britain and Ireland), Richard Harrington (Rothamsted Research), Karen Haysom (Bat Conservation Trust), John Wilkinson (Amphibian and Reptile Conservation Trust), Janet Simkin (British Lichen society), Paul Lee (British Myriapod & Isopod Group), Craig McAdam (Buglife), Judy Webb (Dipterists’ Forum), Mark Stevenson (Defra), David Allen (Natural Resources Wales), Caryn Le Roux (Welsh Government),Sallie Bailey (Forestry Commission), Louise Bond (Scottish Environmental Protection Agency), Alice Hiley (Environment Agency), Tom Hunt (Association of Local Environmental Record Centres), Sarah Whild (Manchester Metropolitan University), Andrea Turner (UK Environmental Observation Framework).

## Appendix S2: instructions given to participants in each of the tasks

Below are the instructions given to the participants of each of the tasks. These tasks were designed by a subset of the authors (MJOP, SEN, IGH, JP and DBR).

### Task 1.

A small group (MJOP, SEN, IGH, JP and DBR) produced a list of proposed attributes and circulated these to the invited participants by email. Wording for task 1.

*Below is a list of aspects that are potentially needed to monitor change in the ‘state of the environment’. We doubt that we have been fully comprehensive, so we would like you to add as many (or as few) additional aspects that you think should be included. If you want to make comments on aspects that we have included in the list then please do so. Once we have received all your responses, we will finalise the list ready for the next task.*

The attributes (Fig. 2; Appendix S3) were then presented to the participants.

### Task 2

Wording for task 2.

*Rank the statements that represent the 10 most important gaps or opportunities in biological recording, based on your perspective.*

*This is the main question in the survey. The statements are those that have already been identified as potentially important by a prior survey.*

*We suggest that you make this a two-stage process:*

*1) Select 'N/A' for the statements that you consider as least important to you, until you are left with 10 or fewer statements. (We recognise that many people will think that most of the statements are important, but we would like you to leave only your top 10 to help us identify priorities for supporting biological recording.)*

*2) Rank the remaining statements from 1 up to a maximum of 10, where 1 is the most important, and 10 the least important. (If you think that only 5 statements are relevant, just rank these 1-5 and apply N/A to the remainder.)*

*(We recognise that your answers may not represent the official view of an organisation or scheme. At all times we will consider your answers to be your personal view and we will not link your identity to your responses.)*

[The statements (Appendix S3) were then presented in an order that was randomised for each participant. Note that we worded this about ‘biological recording’ because this was the term that would be most accessible to the widest range of respondents. Note that the ranks were reversed when scoring for the analysis.]

### Task 3

Wording for task 3.

*Please can you rank the following statements from 1 (the most fundamental needs to monitor change) to 25 (the most aspirational aspects to monitor change).*

*Below are the statements that you saw in task 2 (slightly re-worded for this task). At the workshop we will rank the statements from the most fundamental (e.g. core to a monitoring scheme, the first thing you need in place) to the most aspirational (i.e. would be ‘nice to have’ but potentially complex to put in place and therefore hard to achieve), in regards to monitoring change. We would like to have your opinions on their ranking in advance of the workshop, so we can summarise the responses as a starting point for discussion during the workshop.*

*Please note:*

*1. For this question we are not concerned about their importance per se, we are interested in how fundamental or aspiration they are to monitoring change. We asked about their importance in task 2, before Christmas, and we will present the results of the importance of these gaps/opportunities for discussion at the workshop.)*

*2. You only need to do this ordering roughly, so don't agonise over the precise order. We will summarise the results to use a starting point for our discussion at the workshop.*

*Please remember that this task is a precursor to the workshop. A summary of these results will form the starting point for discussions at the workshop. There will be opportunity to discuss these needs/opportunities in detail at the workshop.*

[The statements (Appendix S3) were then presented in an order that was randomised for each participant.]

## Appendix S3

**Table S1.** The final list of attributes and how they changed through the tasks.

| Final rank*† | Final text of attribute | Summary text of attribute | Task 3, part 1 | Average rank from task 3, part 1 † | Task 2 | Task 1 |
| --- | --- | --- | --- | --- | --- | --- |
| 1 | Articulate the objectives of monitoring | Articulate objectives |  |  |  |  |
| 2 | There is **standardised methodology** and protocols to ensure consistency | Standardised methodology | There is standardised methodology and protocols to ensure consistency | 6.9 | Standardised methodology and protocols are required to ensure consistency and the recording of absences | We need to be able to assess the abundance of species |
| 3 | There are **suitable field sampling methods** that are accurate/efficient | Suitable field sampling methods | There are suitable field sampling methods that are accurate/efficient | 7.6 | Suitable field sampling methods are required, or need to be developed for increased accuracy/efficiency | Suitable field sampling methods are required |
| 4 | There are sufficient **contributors** | Sufficient contributors | There are volunteer contributors | 8.8 | More contributors need to be recruited overall | More contributors need to be recruited |
| 5 | There are suitable and accessible **identification guides** | Identification guides | There are suitable and accessible identification guides | 11.3 | Suitable and accessible identification guides are needed  [added after task 1] |  |
| 6 | There is **national or regional co-ordination** | National/regional co-ordination | There is national co-ordination and support | 9.8 | Better national co-ordination and support is required | 1. A regional co-ordination network is needed 2. Better national co-ordination is required   [combined after task 1] |
| 7 | There are **data systems** (e.g. online) for efficient data capture and storage | Data entry systems | There are data entry systems (e.g. online) for efficient data capture | 9.8 | Improved data entry systems (e.g. online) are needed for improved efficiency | Improved data entry systems (e.g. online) are needed for improved efficiency |
| 8 | There are **quality assurance checks** undertaken in order to ensure the accuracy of the records | Quality assurance of data | There are quality assurance checks undertaken in the field in order to ensure the accuracy of the records | 15.2 | Quality assurance checks in the field need to be implemented (or improved) in order to ensure the accuracy of the records | Field-based quality assurance checks need to be implemented (or improved) |
| 9 | There is appropriate **feedback** to participants on survey results and findings | Feedback to participants | There is feedback to participants on survey results and findings | 9.1 | Feedback to participants on survey results and findings need to be improved | Feedback to participants on survey results and findings need to be improved |
| 10 | There are sufficient contributors with **specialist knowledge** of their taxa | Sufficient specialists | There are sufficient contributors with specialist knowledge of their taxa | 9.4 | More people with specialist knowledge of their taxa are required | More people with specialist knowledge of their taxa are required |
| 11 | There are appropriate **analytical/statistical approaches** to measure trends from monitoring data | Statistical approaches | There are appropriate analytical/statistical approaches to measure trends from monitoring data | 11.9 | New analytical/statistical approaches are needed to measure trends from monitoring data | New analytical/statistical approaches are needed to measure trends from monitoring data |
| 12 | There is good **retention of contributors** | Retention of contributors | There is good retention of contributors and their expertise is enhanced over time (including through the provision of mentoring, support and training) | 10.0 | Current contributors need to be retained and their expertise enhanced (including through the provision of mentoring, support and training)  [added after task 1] |  |
| 13 | Mentoring, t**raining and support** for contributors is provided | Contributor training and support | Training and support for potential contributors is provided | 10.1 | Training and support for potential contributors needs to be provided | Potential contributors to recording schemes require formal training before contributing |
| 14 | There is access to **analytical expertise** to measure trends from monitoring data | Analytical expertise | There is access to analytical expertise to measure trends from monitoring data | 12.8 | Access to analytical expertise is needed to measure trends from monitoring data | Access to analytical expertise is needed to measure trends from monitoring data |
| **15** | **Change is reported** *at appropriate intervals* | Change is reported | Change is reported over time (but not annually) | 10.9 | Need to be able to report change over time (but not annually)  [added after task 1] |  |
| 16 | There is a **scientific scheme design** (such as stratified or randomised site selection) for statistical rigour | Scientific sampling design | There is a scientific scheme design (such as stratified or randomised site selection) for statistical rigour | 12.8 | There needs to be a scientific scheme design (such as stratified or randomised site selection) for statistical rigour | A more robust scheme design is required for statistical rigour |
| 17 | There are simple ways for **everyone to report** widespread/common/easily-identified species | Simple reporting for all | There are simple ways for everyone to report widespread/common/easily-identified species | 13.8 | Simple ways for everyone to report widespread/ common/easily identified species need to be developed  [added after task 1] |  |
| 18 | The results of monitoring schemes are **widely disseminated** | Dissemination of results | The results of monitoring schemes are widely disseminated | 14.1 | The results of monitoring schemes should be widely disseminated | The results of monitoring schemes should be widely disseminated |
| 19 | Examples of **best practice** are identified and shared between schemes and organisations | Sharing best practice | Examples of best practice are identified and shared between schemes and organisations | 15.9 | Examples of best practice should be identified and shared between schemes and organisations  [added after task 1] |  |
| 20 | ‘Important’ or **‘indicator’ species** have been identified | Identify indicator species | ‘Important’ or ‘indicator’ species have been identified | 16.4 | ‘Indicator’ species need to be identified  [added after task 1] |  |
| 21 | There is coverage **widely** across the country/region, e.g. covering remote and well-populated areas | Wide coverage by contributors | There are contributors spread widely across the country, e.g. covering remote and well-populated areas | 13.8 | More contributors need to be recruited to places that are poorly recorded  [added after task 1] |  |
| 22 | Recorders collect **supplementary data** (such as characteristics of the habitat, soil or weather) | Record supplementary data | Recorders collect supplementary data (such as characteristics of the habitat, soil or weather) | 16.8 | Recorders should collect supplementary data (such as characteristics of the habitat, soil or weather)  [added after task 1] |  |
| 23 | There is extra effort on **priority species and habitats** | Important location focus | There is effort focussed on 'important' locations (for example, priority habitats or protected sites) | 17.5 | Effort should be focussed on 'important' locations (for example, priority habitats or protected sites)  [added after task 1] |  |
| 24 | There are systems for **electronically capturing data** in the field | Capturing data in field | There are systems for electronically capturing data in the field | 19.9 | Improved systems for capturing data in the field are needed | Improved systems for capturing data in the field are needed |
| 25 | Change is reported on an **annual** basis | Change reported annually | Change is reported on an annual basis | 21.2 | Need to be able to report change on an annual basis | We need to be able to report change on an annual basis |
|  |  |  | There is effort focussed on ‘important’ species (typically ‘indicators’, but could include ecosystem ‘keystones’ or species important for human wellbeing).  [omitted during workshop] |  | Effort should be focussed on ‘important’ species (typically ‘indicators’, but could include ecosystem ‘keystones’ or species important for human wellbeing) | We should prioritise the monitoring of taxa with relevance to ecosystem functioning |
|  |  |  |  |  |  | Only the rarest species need to be monitored [omitted after task 1] |
|  |  |  |  |  |  | Only the most abundant species need to be monitored [omitted after task 1] |

* The final rank was a consensus reached with the workshop participants using the initial aggregated ranks from task 3 as a starting point. Note that most attributes were retained in the same order as the aggregated ranks from task 3, but some attributes changed in response to discussion and consensus.

† Attributes were ranked from one (the most elemental, i.e. most essential for monitoring change) to 25 (the most aspirational, i.e. nice to have). The aggregated rank is averaged over all 17 respondents.

## Appendix S4: Comparison of the data set with all respondents to that with only the invited participants

**Table S4.** Comparison of the data set with all respondents, as presented in the main paper, and with the invited participants only. The patterns are broadly similar between the two.

| Descriptor | Full data set | With invited participants only | Excluding respondents who were also authors |
| --- | --- | --- | --- |
| % variance explained by PC1 | 13.3% | 16.3% | 13.9% |
| % variance explained by PC2 | 8.9% | 12.9% | 9.2% |
| % variance explained by each of the remaining PCs | <7.6% | <8.8% | <7.5% |
| Number of clusters identified with the ensemble in ‘NbClust’ | 2 | 2 or 15 | 2 or 3 |
| Maximum Gap_k_ statistic | 2 clusters | 5 clusters is the global maximum but 3 is a local maximum | 2 clusters |
| Summary description of attribute* | Loading on PC1 for all data (rank) | Loading on PC1 for invited participants only (rank) | Loading on PC1 for non-authors only (rank) |
| Standardised methodology | -0.5 (1) | -0.31 (2) | -0.51 (1) |
| Scientific sampling design | -0.27 (2) | -0.22 (5) | -0.27 (2) |
| National/regional coordination | -0.2 (3) | -0.11 (7) | -0.19 (3) |
| Suitable field sampling methods | -0.17 (4) | -0.09 (9) | -0.19 (4) |
| Change is reported | -0.13 (5) | -0.36 (1) | -0.1 (7) |
| Statistical approaches | -0.12 (6) | -0.26 (3) | -0.13 (5) |
| Analytical expertise | -0.1 (7) | -0.23 (4) | -0.11 (6) |
| Data entry systems | -0.08 (8) | -0.1 (8) | -0.07 (10) |
| Important species focus | -0.07 (9) | 0.23 (22) | -0.09 (8) |
| Quality assurance of data | -0.07 (10) | 0.05 (16) | -0.09 (9) |
| Change reported annually | -0.04 (11) | -0.06 (10) | -0.02 (12) |
| Record supplementary data | -0.03 (12) | -0.06 (11) | -0.01 (14) |
| Simple reporting for all | -0.02 (13) | -0.03 (12) | -0.03 (11) |
| Capturing data in field | -0.01 (14) | 0.05 (15) | 0 (15) |
| Dissemination of results | 0.01 (15) | 0.05 (17) | 0.01 (17) |
| Identify indicator species | 0.01 (16) | 0.08 (19) | -0.01 (13) |
| Important location focus | 0.01 (17) | 0 (13) | 0.01 (16) |
| Sharing best practice | 0.03 (18) | -0.16 (6) | 0.05 (19) |
| Feedback to participants | 0.05 (19) | 0.06 (18) | 0.04 (18) |
| Identification guides | 0.14 (20) | 0.16 (21) | 0.14 (21) |
| Sufficient specialists | 0.15 (21) | 0.09 (20) | 0.12 (20) |
| Better spatial coverage | 0.23 (22) | 0.04 (14) | 0.26 (22) |
| Wide coverage by contributors | 0.29 (23) | 0.3 (23) | 0.31 (23) |
| Contributor training and support | 0.38 (24) | 0.46 (25) | 0.36 (24) |
| Retention of contributors | 0.46 (25) | 0.37 (24) | 0.46 (25) |
